# Do colorectal cancer patients diagnosed as an emergency differ from non-emergency patients in their consultation patterns and symptoms? A longitudinal data-linkage study in England

**DOI:** 10.1038/bjc.2016.250

**Published:** 2016-08-18

**Authors:** C Renzi, G Lyratzopoulos, T Card, T P C Chu, U Macleod, B Rachet

**Affiliations:** 1 Health Behaviour Research Centre, Department of Epidemiology and Public Health, University College London, WC1E 6BT London, UK; 2 Cancer Survival Group, Department of Non-communicable Disease Epidemiology, London School of Hygiene and Tropical Medicine, WC1E 7HT London, UK; 3 Cambridge Centre for Health Services Research, University of Cambridge, CB2 0SR Cambridge, UK; 4 Division of Epidemiology and Public Health, University of Nottingham, NG5 1PB Nottingham, UK; 5 Nottingham Digestive Diseases Centre Biomedical Research Unit, University of Nottingham, NG7 2UH Nottingham, UK; 6 Division of Child Health, Obstetrics and Gynaecology, University of Nottingham, NG7 2UH Nottingham, UK; 7 Hull York Medical School, University of Hull, HU6 7RX Kingston upon Hull, UK

**Keywords:** symptomatic presentations, primary care, emergency diagnosis, colorectal cancer, data-linkage study

## Abstract

**Background:**

More than 20% of colorectal cancers are diagnosed following an emergency presentation. We aimed to examine pre-diagnostic primary-care consultations and related symptoms comparing patients diagnosed as emergencies with those diagnosed through non-emergency routes.

**Methods:**

Cohort study of colorectal cancers diagnosed in England 2005 and 2006 using cancer registration data individually linked to primary-care data (CPRD/GPRD), allowing a detailed analysis of clinical information referring to the 5-year pre-diagnostic period.

**Results:**

Emergency diagnosis occurred in 35% and 15% of the 1029 colon and 577 rectal cancers. ‘Background' primary-care consultations (2–5 years before diagnosis) were similar for either group. In the year before diagnosis, >95% of emergency and non-emergency presenters had consulted their doctor, but emergency presenters had less frequently relevant symptoms (colon cancer: 48% *vs* 71% (*P*<0.001); rectal cancer: 49% *vs* 61% (*P*=0.043)). ‘Alarm' symptoms were recorded less frequently in emergency presenters (e.g., rectal bleeding: 9 *vs* 24% (*P*=0.002)). However, about 1/5 of emergency presenters (18 and 23% for colon and rectal cancers) had ‘alarm' symptoms the year before diagnosis.

**Conclusions:**

Emergency presenters have similar ‘background' consultation history as non-emergency presenters. Their tumours seem associated with less typical symptoms, however opportunities for earlier diagnosis might be present in a fifth of them.

According to international data, between 14 and 33% of colorectal cancers are diagnosed as emergencies (Gunnarsson *et al*, 2014). Despite some recent progress, in England a diagnosis of cancer following an emergency presentation still occurs in as many as 22% of colorectal cancers, with significant socio-economic inequalities (NCIN, 2015). Emergency presenters are less often treated with curative intent (McArdle and Hole, 2004), even after controlling for stage at diagnosis (McPhail *et al*, 2013), and they have poorer survival (Elliss-Brookes *et al*, 2012; Downing *et al*, 2013). Moreover, emergency presentations are associated with worse patient-reported outcomes (Quality Health, 2014) and disruptions to hospital services (Goodyear *et al*, 2008). Reducing emergency presentations could therefore lead to more efficient and appropriate use of health services, and substantially improve health outcomes.

However, studies examining potentially modifiable risk factors and circumstances surrounding emergency cancer diagnosis are limited (Mitchell *et al*, 2015a). Some studies have shown an increased risk of emergency colorectal cancer diagnosis for women (Abel *et al*, 2015), older (Mitchell *et al*, 2015a) and more deprived people (Raine *et al*, 2010; Mayor, 2012), but the findings are not always consistent. Few studies have examined colon and rectal cancers separately (McArdle and Hole, 2004; Rabeneck *et al*, 2006; Gunnarsson *et al*, 2013, 2014; Abel *et al*, 2015), even though these two cancer sites often have distinct clinical presentations and the prevalence of emergency diagnosis is markedly different (31% for colon and 15% for rectal cancers; Abel *et al*, 2015). Only very limited evidence is available on symptoms and health-care use before emergency cancer diagnosis. According to one Swedish study on colon cancer (Gunnarsson *et al*, 2014) and two UK studies on colorectal cancers diagnosed in London (Sheringham *et al*, 2014) and Exeter (Cleary *et al*, 2007) most patients have seen their doctor during the 6 months before diagnosis, often with non-specific symptoms. Case note reviews within clinical audits (Rubin *et al*, 2011), qualitative studies (Black *et al*, 2015) and patient surveys (Lyratzopoulos *et al*, 2012) have also provided some insights into potential opportunities to diagnose cancer earlier, but they are often limited by participation and recall bias, due to retrospective data collection after patients received a cancer diagnosis.

Some emergency diagnoses can be regarded as unavoidable, such as in the case of cancers with a sudden clinical presentation with minimal or no prior symptoms (Lyratzopoulos *et al*, 2014). Other cases are potentially avoidable and these include: (a) patients who, despite having symptoms, did not seek help promptly due to psycho-social factors or health-care system barriers (in this case public education and removing barriers to health care are necessary); (b) patients who sought help for symptoms, but opportunities were missed due to atypical symptoms, or deficiencies in investigations or other factors. The proportion of patients falling into each of the above categories is unknown.

In order to provide a population-level picture of symptomatic presentations during the months and years before the cancer diagnosis and to identify opportunities for reducing emergency diagnoses we used national cancer registration data individually linked to clinical data prospectively collected in primary care within the Clinical Practice Research Datalink (CPRD—previously GPRD). CPRD is a large database of anonymised primary-care records from over 600 general practices. It is validated and extensively used for epidemiological research and is considered to be representative of the UK population (Khan *et al*, 2010; Dregan *et al*, 2012; Tsang *et al*, 2013; Chu *et al*, 2015; Din *et al*, 2015). The database is particularly suited for the present study as it provides details on the medical history of patients, including prospectively recorded patient-level information on each episode of illness, symptom occurrences, all significant clinical contacts, diagnoses and abnormal test results.

The objectives of our study were to examine patterns of presentation in primary care with symptoms/signs potentially related to colon and rectal cancer during the years and months before the cancer diagnosis. In particular, we aimed to compare patients with a cancer diagnosis following an emergency presentation with patients diagnosed after non-emergency referrals, taking socio-demographic factors into account, in order to identify opportunities for reducing emergency presentations. This will be useful for providing evidence that can inform the development of interventions aimed at reducing emergency cancer diagnosis, and for improving quality of care and cancer outcomes.

## Materials and methods

### Study sample and data sources

We have conducted a cohort study using data from the population-based National Cancer Registry linked to CPRD/GPRD data for patients with an incident colon or rectal cancer (ICD10 codes C18 and C19–C20, respectively). We included cancers diagnosed in England in 2005 and 2006, as this represents the latest cohort with linked CPRD data available to us, providing information on signs and symptoms for up to 10 years before the cancer diagnosis (Ethics approval: ISAC-Protocol 08_031R; NHS Health Research Authority Confidentiality Advisory Group (PIAG 1–05(c)/2007)). The present study focused on the 5-year pre-diagnostic period, as an initial examination of consultation patterns going back to 10 years showed no relevant variations in consultation rates 5–10 years before the cancer diagnosis.

Inclusion criteria were age 25 years or older, no previous diagnosis of cancer at any site, at least 1 year of CPRD records before cancer diagnosis. Individuals with a previous cancer diagnosis were not included as they probably have different help-seeking behaviour and health-care use (due to increased cancer awareness and possibly regular follow-up visits) compared with primary-care patients overall. Doctors might also be more prone to consider cancer as a possible explanation for symptoms presented by these patients. This subgroup merits to be examined separately, but this was not possible in the present study due to small numbers.

The CPRD includes an ‘up-to-standard' date, indicating when the data meet pre-defined quality criteria in over 80 variables. We included only records meeting these criteria in order to reduce the risk of missing or inaccurate data.

**Figure 1:**
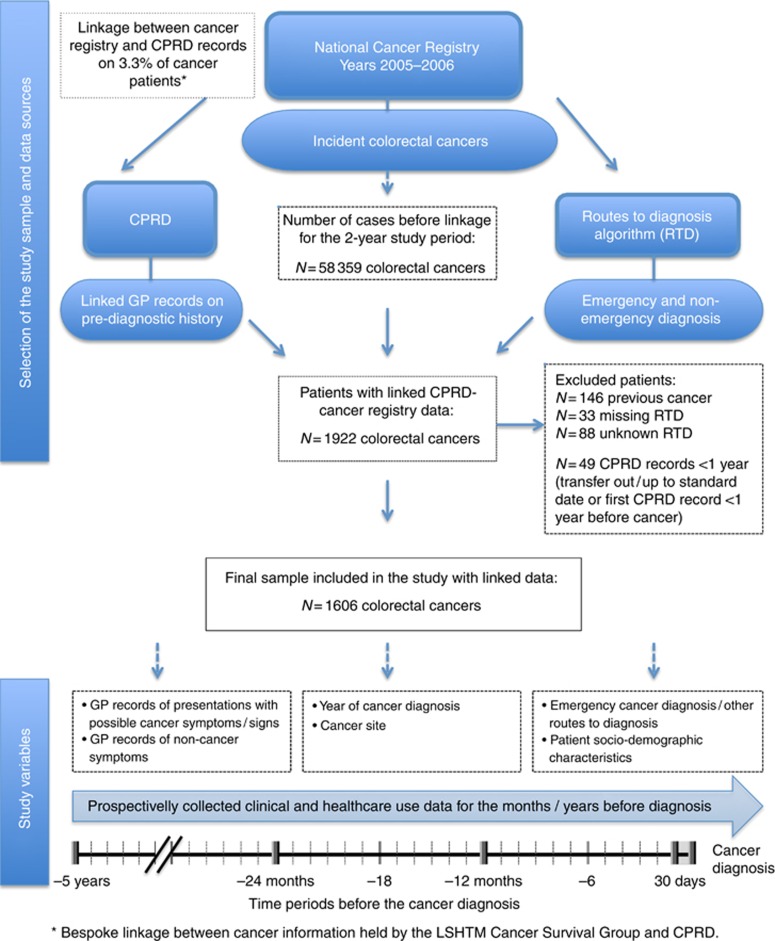
**Study sample selection and data sources.**

Of the 58 359 incident colon and rectal cancer patients identified in the National Cancer Registry, 1922 patients were linked to CPRD (3.3%). This was in line with expectations, considering that about half of all GP practices included in CPRD (covering ∼7% of the population in England) participate in the data-linkage scheme. Non-participation in the linkage scheme is mostly due to non-response rather than active refusal. After applying the study-exclusion criteria a total of 1606 patients were included in the final study sample (Figure 1). On average, each GP practice contributed to 8 cancer patients over the total study period.

### Variable definitions

Our outcome of interest was an emergency cancer diagnosis, defined according to the ‘routes to diagnosis' algorithm based on several routine data sets and provided by NCIN (Elliss-Brookes *et al*, 2012; NCIN, 2013). In particular, an emergency diagnosis is defined as a diagnosis of cancer following presentation to an Accident and Emergency Unit, or following a GP emergency referral or following emergency pathways for in/out-patients (Elliss-Brookes *et al*, 2012; NCIN, 2013). Non-emergency cancer diagnoses include routine GP referrals, 2-week wait GP referrals (introduced in 2000 to allow GPs to refer suspected cancer patients urgently, so that they can see a specialist within 2 weeks), elective inpatient/outpatient and screening. For the purpose of our study focusing on emergency presentation, and similarly to previous research (McPhail *et al*, 2013), after an initial description of the different routes we have grouped patients into two categories: emergency and non-emergency cancer patients (the latter including all the non-emergency routes).

Our main explanatory variables were signs and symptoms recorded in primary care prior to the cancer diagnosis. On the basis of the published literature (Sheringham *et al*, 2014; Din *et al*, 2015) and guidelines (NICE Guidelines, 2015), we have operationally defined signs/symptoms potentially relevant for colorectal cancer. Our preliminary list has been reviewed by clinical experts and a final list has been compiled (Supplementary Material 1). Examples of relevant signs/symptoms are as follows: rectal bleeding, change in bowel habits, palpable rectal mass, iron-deficiency anaemia, abdominal pain and weight loss. Read codes for relevant symptoms have been identified and applied to records in CPRD (Supplementary Material 1). The Read codes included in the final list are as comprehensive as possible, considering that different codes can be used for similar symptoms (e.g., 16 different codes were included for identifying patients with diarrhoea). It was based on codes used in previous studies (Sheringham *et al*, 2014) and further expanded following a detailed revision by clinical experts, as well as an examination of the data and the Read Code hierarchy (see Supplementary Material 2 for details on the development of the list of signs/symptoms).

In order to account for patient characteristics we also examined age, gender and deprivation, based on the income domain of the Index of Multiple Deprivation for England (Department for Communities and Local Government, 2008, The English Indices of Deprivation, 2007 London).

### Statistical analysis

We initially described the socio-demographic characteristics, number, type and timing of symptoms before the cancer diagnosis separately for patients with emergency and non-emergency presentation. Colon and rectal cancers were examined separately throughout.

We then examined predictors of emergency diagnosis in univariable analyses, and assessed significance using *χ*^2^-test (or test for trend for ordered categorical variables). To compare the median number of consultations for any reason >24 months before cancer diagnosis in emergency and non-emergency presenters we used the Wilcoxon rank-sum test. Similarly, consultations for any reason during the year before cancer diagnosis have been examined. As events occurring shortly before diagnosis might be related to the diagnostic episode itself, rather than represent opportunities for earlier diagnosis, the 30 days before diagnosis have been examined separately throughout.

We examined the proportions of patients with at least one relevant symptom and with each specific symptom in different time periods before the cancer diagnosis (Figure 1) and we compared these proportions between emergency and non-emergency presenters using *χ*^2^ statistics. Consultation rates with relevant symptoms over the 5-year time period have been calculated and divided in bi-monthly, six-monthly and yearly time periods, in order to examine changes in consultation rates over time. We have examined whether consultation rates with relevant symptoms significantly varied by emergency presentation status using Poisson regression. The models included age, sex and deprivation, and were fitted for each time period separately, focusing on the 6 months and the year before diagnosis, as well as 13–24 months and 25–36 months before diagnosis. Random effects were included to account for patient-level clustering of symptomatic presentations.

Finally, multivariable logistic regression was used for examining the risk of emergency diagnosis according to type and timing of symptoms, and taking into account the number of consultation for any reason during the year before diagnosis and socio-demographic characteristics. The final model included variables thought *a priori* to be potentially important explanatory variables based on previous evidence and clinical reasoning (i.e., socio-demographic factors and number of consultations), and the specific symptoms that were associated with emergency presentation at univariable analysis. As observations within GP practices are not independent (mean 8 observations per practice, range 1–26) robust standard errors were calculated.

Interactions between the variables included in the final model were examined (e.g., interaction between each symptom recorded the year before diagnosis and the same symptom in earlier time periods, and between symptoms and socio-demographic factors), but power was limited due to sparse data.

STATA14 software (Stata Corp, College Station, TX, USA) was used for statistical analyses.

## Results

### Socio-demographic characteristics and emergency cancer diagnosis

Among the 1606 included patients 52% of colon and 58% of rectal cancer patients were men and the median age was 74 years (interquartile range (IQR) 65–81) and 73 years (IQR 63–80). The demographic characteristics of our study cohort were comparable with those of colorectal cancer patients in the 2005 and 2006 Cancer Registry not linked to CPRD. Among the study cohort, 35% of colon and 15% of rectal cancer patients had an emergency cancer diagnosis.

An emergency diagnosis was more frequent in women (*P*=0.04 for both colon and rectal cancers), and older patients, particularly ages 80 years and above (*P*=0.04 for colon and *P*=0.003 for rectal cancers); it was also more frequent among socio-economic deprived patients for rectal cancers only (*P*<0.001; Table 1).

### Consultations for any reason before the cancer diagnosis

The great majority of the study cohort had primary-care information for the whole of the 5-year pre-diagnostic period, with only 2% of the cohort having primary-care records covering <2 years before diagnosis.

GP consultation rates per year for any reason during the time period 2–5 years before diagnosis were not significantly different between diagnostic routes, with 88% of both colon and rectal cancer patients having seen their GP at least once a year (Table 2); the median number of consultations per year was 5 (IQR 2–10) for non-emergency and emergency colon cancer patients; and 5 for both non-emergency and emergency rectal cancer patients (IQR 2–9 and 2–12, respectively). Consultations for any reason increased for all patients during the 13–24 months before diagnosis and even more so during the year before diagnosis. Specifically, as shown in Table 2, during the year before diagnosis consultations were significantly higher for non-emergency colon cancer patients (median 12; IQR 7–18) compared with emergency presenters (median 10; IQR 5–19). Non-emergency rectal cancer patients had fewer consultations during the year before diagnosis (median 9; IQR 5–13) compared with emergency presenters (median 12; IQR 6–20). Only a small minority of patients (2.4 and 3.1% of colon and rectal cancers, respectively) have never seen their GP during the year before diagnosis, with minimal differences between emergency and non-emergency presenters.

### Consultations for relevant symptoms before the cancer diagnosis

The majority of patients had at least one consultation with a relevant symptom recorded during the year before diagnosis (80 and 84% among colon and rectal cancers, respectively; Table 3). However, the proportion of patients with at least one relevant symptom was significantly lower in emergency compared with non-emergency presenters, particularly when excluding the 30 days before diagnosis (colon: 48 *vs* 71%, *P*<0.001; rectal cancers: 49% *vs* 61%, *P*=0.043).

**Figure 2:**
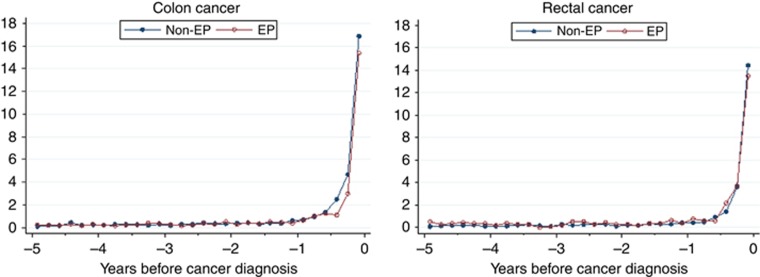
**Rates of consultations with relevant symptoms for emergency (EP) and non-emergency (non-EP) presenters: bi-monthly rates (per 100 person-years).**

‘Background' consultation rates with a potentially relevant symptom were very low and remained stable during the 5-year period up until ∼12–17 months before diagnosis (Figure 2). For both colon and rectal cancer patients, consultation rates increased markedly during the year before diagnosis, particularly during the last 6 months, with no apparent differences by emergency presentation status. Using Poisson regression and controlling for socio-demographic variables showed that consultation rates during the year before diagnosis were not significantly different for emergency *vs* non-emergency presenters (incidence rate ratio (IRR) for colon cancer=0.86; 95% CI 0.7–1.1; *P*=0.182; rectal cancer=1.26; 95% CI 0.9–1.8; *P*=0.210). However, when restricting to the last 6 months before diagnosis, emergency presenters with colon cancer had a significantly lower consultation rate (IRR=0.76; 95% CI 0.6–0.9; *P*=0.039).

### Specific relevant symptoms before the cancer diagnosis

The potentially relevant symptoms/signs most frequently recorded during the year before diagnosis (excluding the 30 days) were abdominal pain (25.1%), anaemia (19.2%), diarrhoea (9.9%) and rectal bleeding (9.4%) among colon cancer patients, and rectal bleeding (21.5%), change in bowel habits (11.6%), diarrhoea (12%) and abdominal pain (9.4%) in rectal cancers patients (Table 3). However, symptoms were different according to emergency presentation status, particularly for colon cancers where ‘red-flag symptoms' were more prevalent among non-emergency presenters compared with emergency presenters: anaemia (23.2 *vs* 11.9% *P*<0.001), rectal bleeding (12.6 *vs* 3.6% *P*<0.001) and change in bowel habits (6.7 *vs* 3.3% *P*=0.022). Among rectal cancer patients, only rectal bleeding was significantly more prevalent in non-emergency presenters (23.7 *vs* 9.2% *P*=0.002). Overall, 31.8% of colon cancer and 36.4% of rectal cancer patients had at least one of the above-mentioned ‘red-flag' symptoms recorded between 30 days and 12 months pre-diagnosis. Non-emergency presenters had a higher prevalence of at least one red-flag symptom compared with emergency presenters (colon: 39.5 *vs* 17.5% *P*<0.001; rectal cancer: 38.8 *vs* 23% *P*=0.005).

Among patients with at least one relevant symptom, 47% of colon and 43% of rectal cancer patients had multiple visits with the same symptom during the period between 30 days and 12 months pre-diagnosis, without statistical evidence for variation in this proportion by emergency presentation status (data not shown).

Examining potentially relevant symptoms recorded in more distant years (i.e., between 25–60 months pre-diagnosis) has shown that emergency rectal cancer patients had more frequently a past record of anaemia (8.1 *vs* 2.0% *P*=0.002) and change in bowel habits (2.3 *vs* 0.4% *P*=0.050) compared with non-emergency presenters. Among colon cancer patients, emergency presenters had less frequently a past record of rectal bleeding (1.7 *vs* 3.9% *P*=0.049) than non-emergency presenters. Overall, the prevalence of at least one red-flag symptom was much lower during the more distant time periods compared with the year before diagnosis (e.g., 5.9 and 4.7% among colon and rectal cancers, respectively, 13–24 months before diagnosis) without apparent differences by emergency presentation status.

### Multivariable analysis examining the effect of symptomatic presentations and socio-demographic factors on emergency cancer diagnosis

Multivariable logistic regression analysis, including socio-demographic factors and relevant symptoms into the model, has shown that in the period from 30 days to 12 months pre-diagnosis the risk of emergency colon cancer diagnosis was significantly lower for patients with a record of anaemia (OR=0.38; 95% CI 0.3–0.6), change in bowel habits (OR=0.47; 95% CI 0.3–0.9) or rectal bleeding (OR=0.22; 95% CI 0.1–0.4; Table 4). On the other hand, emergency diagnosis was more likely in women (OR=1.37; 95% CI 1.0–1.8) and people aged 80 years and older (OR=1.84; 95% CI 1.2–2.7), independently of symptom history. For rectal cancers, only rectal bleeding during the year before diagnosis was associated with a lower risk of emergency presentation (OR=0.25; 95% CI 0.1–0.6). Socio-economic deprivation was associated with a higher risk of emergency presentation for rectal cancer, independently of symptoms (e.g., most deprived category OR=3.47; 95% CI 1.5–8.0). Increasing number of consultations for any reason during the year before diagnosis somewhat increased the risk of emergency presentation for rectal cancer (OR=1.03; 95% CI 1.0–1.1). This was also confirmed after excluding outliers, that is, patients with a very high number of consultations (upper 5th percentile, corresponding to >32 consultations during the year before diagnosis; data not shown). There was some indication that change in bowel habits (OR=12.0; 95% CI 1.6–92.1) and anaemia (OR=2.67; 95% CI 0.8–8.9) recorded 25–60 months pre-diagnosis might increase the risk of emergency rectal cancer but confidence intervals were wide, reflecting the small number of individuals with such records.

## Discussion

### Main findings

Linked cancer registration and primary-care data allowed for a detailed description of clinical presentations in primary care before a cancer diagnosis, comparing patients diagnosed as an emergency with those diagnosed through non-emergency routes. The longitudinal data have shown that consultation patterns between 12 months and up to 5 years pre-diagnosis were very similar in emergency and non-emergency presenters. Consultation rates increased significantly in the last months before diagnosis independently of the diagnostic route. Emergency presenters are not a uniform category and they can be divided into different groups according to their consultation history. Only a very small minority of emergency presenters have never consulted for any reason during the year before diagnosis. However, less than half of emergency presenters have clinical records of relevant cancer symptoms, which is significantly lower than among non-emergency presenters. Nevertheless, approximately a fifth of emergency presenters had typical ‘alarm' symptoms and 16% had 3 or more consultations with relevant symptoms, suggesting possible opportunities for earlier diagnosis.

### Comparison with other studies and possible explanations for our findings

Our findings are in line with previous studies showing that most emergency presenters have primary-care consultations during the months before the cancer diagnosis (Cleary *et al*, 2007; Gunnarsson *et al*, 2014; Sheringham *et al*, 2014). Our results are also in agreement with a study based on direct record reviews reporting that 60% of emergency colorectal cancer patients had relevant symptoms 1 month or more before diagnosis (Cleary *et al*, 2007).

Abdominal pain and rectal bleeding are the most frequent symptoms among colon and rectal cancer patients, respectively, (Hamilton *et al*, 2013) and similarly to previous research, we found a lower risk of emergency presentation for patients with rectal bleeding, a well-recognised symptom of colorectal cancer (Cleary *et al*, 2007; Gunnarsson *et al*, 2014; Sheringham *et al*, 2014). Earlier research highlighted an increased risk of emergency diagnosis in case of abdominal pain and constipation (Sheringham *et al*, 2014), diarrhoea and weight loss (Cleary *et al*, 2007). Concordantly, we found that these symptoms/signs were all associated with emergency diagnosis, but only when focusing on the last 30 days before diagnosis. These symptoms/signs can be an indication of progression towards occlusion, which may explain their higher prevalence among emergency presenters shortly before diagnosis.

Anaemia and change in bowel habits, typical red-flag symptoms generally leading to prompt investigations, were also associated with a lower risk of emergency colon cancer diagnosis. Anaemia and change in bowel habits recorded 2–5 years pre-diagnosis might increase the risk of emergency presentation, but sparse data limited our analyses. These sign/symptoms might have been initially dismissed as benign and subsequently neglected by patients and/or doctors, as suggested by previous research (Mitchell *et al*, 2015b; Renzi *et al*, 2016).

Importantly, our study has highlighted that during the year before diagnosis one in five emergency presenters had at least one red-flag symptom, suggesting opportunities for earlier diagnosis in these cases. Opportunities are probably even more prevalent, considering that symptoms are likely to be under-recorded, as suggested by the fact that one out of three non-emergency presenters had no relevant symptom recorded the year before diagnosis.

On the basis of international data, missed opportunities can occur in 1 out of 3 colorectal cancer patients, with older age, comorbidities and belonging to ethnic minority groups increasing the risk (Singh *et al*, 2009). Multiple factors are often implicated, including patient, doctor and health-care system factor (Lyratzopoulos *et al*, 2015).

We found that between 16 and 22% of colon and rectal cancer patients had three or more consultations with relevant symptoms during the year before diagnosis, which is consistent with UK audit data (Rubin *et al*, 2011). Our study has highlighted that consultation rates overall and consultations with relevant symptoms increased substantially during the months before diagnosis among emergency and non-emergency presenters. In the case of rectal cancers the risk of emergency presentation increased with increasing number of consultations for any reason. This is in contrast with previous studies (Sheringham *et al*, 2014), but differences between colon and rectal cancers, and changes in the patterns of symptoms during the last 30 days before diagnosis were previously not taken into account.

Our study has shown that in some cases despite specific symptoms, cancer was only diagnosed after emergency presentation, and this more likely occurred in some subgroups. Women, older and more deprived individuals have been previously shown to be at higher risk of emergency diagnosis (Raine *et al*, 2010; Mayor, 2012; Abel *et al*, 2015; Mitchell *et al*, 2015a), and our data indicate that these subgroups are at higher risk independently of symptomatic presentations. Further research is warranted to understand the role played by patient factors (e.g., missed follow-up visits), health-care factors (e.g., delays in diagnostic work-up, previous borderline/normal test results), as well as clinical and tumour factors complicating the diagnosis (comorbidities, proximal cancers). For example, in-depth quantitative and qualitative studies would be necessary examining the role of comorbidities (Barnett *et al*, 2012; Mitchell *et al*, 2015b), their effect on patients' interpretation and reporting of cancer symptoms, as well as their effect on doctors' decision-making regarding differential diagnosis, referrals and testing.

The bowel cancer screening programme started in 2006 in England and limited evidence is available on a possible positive effect of screening and other early diagnosis/cancer awareness initiatives (NICE Guidelines, 2015; Be Clear on Cancer, 2016) on emergency presentations (Goodyear *et al*, 2008; Mansouri *et al*, 2015). Due to socio-economic differences in screening uptake (von Wagner *et al*, 2011), inequalities in emergency presentations and cancer outcomes may, however, persist. Dedicating particular attention to higher-risk groups will therefore remain paramount.

### Strengths and limitations

The strengths of the study include the use of prospectively recorded population-based data comparing emergency and non-emergency cancer diagnoses defined according to validated methodologies (Elliss-Brookes *et al*, 2012; NCIN, 2013). Thanks to the high quality of the data sources, missing information on routes to diagnosis and socio-demographic characteristics were negligible. Moreover, our study cohort was comparable in terms of demographic characteristics to colorectal cancer patients in the Cancer Registry not linked to CPRD. Our study provided specific clinical insights for colon and rectal cancers regarding the pre-diagnostic period. By simultaneously evaluating the role of symptomatic presentations and patient characteristics we identified subgroups at higher risk of missed opportunities and emergency diagnosis, who could benefit from increased clinical and public health efforts. The study demonstrates the usefulness of linked cancer registration and primary-care data (such as CPRD) for early diagnosis research.

Our study will need to be extended to more recent cohorts of cancer patients with individually linked primary-care data. However, although some changes occurred since the study period in guidelines, clinical practice and patient awareness of symptoms (Moffat *et al*, 2015), the natural history of colorectal cancer and the disease processes determining the occurrence of signs and symptom will not have changed. It is also noteworthy that emergency presentations have remained stable over recent years for rectal cancers with a slight decrease for colon cancers (Abel *et al*, 2015); moreover, socio-demographic inequalities in emergency presentations (Abel *et al*, 2015) and cancer survival (Ellis *et al*, 2012) are still relevant (NCIN, 2015). We have performed sensitivity analyses evaluating whether our results differed for patients diagnosed in 2005 and 2006, which showed that the overall findings were not affected by the year of diagnosis in our sample.

Our results have to be interpreted with caution as the examined symptomatic presentations are based on clinical records and do not fully reflect all symptoms experienced by patients. However, this can be assumed to apply equally to emergency and non-emergency presenters. Moreover, clinical data were recorded prospectively by >200 GP practices before the cancer diagnosis, and emergency and non-emergency patients had similar records regarding their background consultation history.

Although routine data sources may contain inaccuracies, the validity of diagnostic coding and consultation rates in CPRD has been extensively confirmed (Khan *et al*, 2010; Dregan *et al*, 2012). CPRD are electronic versions of case notes and therefore include data reported by patients and considered relevant by doctors. It should be noted that sometimes doctors record clinical information only in free-text format rather than READ codes (Price *et al*, 2016). We did not have access to free-text information, which might have led to an underestimation of symptoms. Interviews with patients/doctors could verify the validity and improve accuracy, but this is beyond the purpose of the present work. Similarly, we lacked data on patient experience which can provide important insights. The possibility of linking CPRD records to patient experience data is an area that would merit future consideration in order to overcome this limitation.

### Implications of findings

This study has shown that emergency presenters have similar ‘background' consultation history as non-emergency presenters and their consultation rates increase markedly the year before diagnosis. Even though their tumours seem associated with less typical symptoms, opportunities for earlier diagnosis might be present in a fifth of them. In order to reduce emergency presentations, multi-disciplinary system-wide approaches are needed (Lyratzopoulos *et al*, 2015) addressing critical points along the diagnostic process, as well as targeting different patient subgroups (Borowski *et al*, 2016). More specifically, our findings underscore the importance of dedicating particular attention to patients consulting more frequently than usual, even if their symptoms are not immediately suggestive of cancer. In these cases, and in particular if patients belong to categories at higher risk of emergency diagnosis, such as the elderly, women and socio-economically deprived individuals, a variety of approaches could be employed. Specifically, these can include more pro-active and systematic symptom elicitation (Birt *et al*, 2014; McLachlan *et al*, 2015) and symptom monitoring ensuring a holistic approach (Mitchell *et al*, 2015b), possibly with the support of alternative health-care providers. Considering that a typical GP will only have around 10 min per appointment (Independent Cancer Taskforce, 2015), a specifically trained nurse could support the GP during the initial diagnostic phases and for subsequent follow-up visits and safety-netting. Pre-booked follow-up visits could be particularly useful for patients belonging to higher-risk groups (Mitchell *et al*, 2015b). Moreover, closer interaction and easier access to specialist advice for GPs would be important, in addition to the development of multi-disciplinary diagnostic centres (Independent Cancer Taskforce, 2015). Clinicians and public education campaigns should not only emphasise the importance of discussing symptoms with the doctor when they first appear, but also encourage and support subsequent monitoring of symptoms facilitating prompt re-evaluation if symptoms do not improve.

Regarding the subgroup of patients presenting with relevant symptoms, more systematic use of safety-netting, and prompt specialist referrals and diagnostic investigations would help to seize the opportunities for earlier diagnosis.

Reducing emergency presentations will allow more efficient and appropriate use of health services, improve patient experience of care and increase survival for cancer patients.

## Figures and Tables

**Table 1:** Diagnosis of colon or rectal cancer following EP by patients' socio-demographic characteristics (univariable analysis)

	**Colon cancer**				**Rectal cancer**			
	**Non-EP**^a^	**EP**	**Total**		**Non-EP**^a^	**EP**	**Total**	
	***N*****=668**	***N*****=361**	***N*****=1029**		***N*****=490**	***N*****=87**	***N*****=577**	
	**%**	**%**	***N***	***P*****-value**^b^	**%**	**%**	***N***	***P*****-value**^b^
**Gender**								
Men	67.8	32.2	537	0.044	87.5	12.5	336	0.041
Women	61.8	38.2	492		81.3	18.7	241	
**Age (years)**								
25–59	67.8	32.2	152	0.041	92.8	7.2	97	0.003
60–69	68.6	31.4	204		85.0	15.0	133	
70–79	69.6	30.4	362		86.6	13.4	216	
80+	55.6	44.4	311		76.3	23.7	131	
**Socio-economic deprivation quintile**								
1 (least deprived)	67.2	32.8	268	0.159	90.9	9.1	143	<0.001
2	63.0	37.0	211		86.4	13.6	125	
3	69.3	30.7	228		87.2	12.8	125	
4	63.4	36.6	205		81.1	18.9	111	
5 (most deprived)	57.3	42.7	117		72.6	27.4	73	
**Geographic region**								
North	66.0	34.0	235	0.780	80.1	19.9	151	0.170
Midlands/East England	62.5	37.5	307		85.3	14.7	177	
London	66.2	33.8	71		82.5	17.5	40	
South	65.9	34.1	416		88.5	11.5	209	

Abbreviation: EP=emergency presentation.

a Non-emergency routes included non-urgent GP referrals (colon cancer: 36% rectal cancer: 45%), ‘two-week wait' GP referrals (colon cancer: 10% rectal cancer: 21%) and elective in-/out-patients (20% for both cancers). Screening accounted only for 0.2% of rectal cancers, as the programme started in 2006.

b *χ*^2^-Test was used for gender and region. Test for trend was used for age and socio-economic deprivation.

**Table 2:** GP consultations for any reason for patients diagnosed with colon or rectal cancer following EP *vs* non-EP

	**Colon cancer**				**Rectal cancer**			
	**Total**	**Non-EP**	**EP**		**Total**	**Non-EP**	**EP**	
	***N*****=1029**	***N*****=668**	***N*****=361**		***N*****=577**	***N*****=490**	***N*****=87**	
	**%**	**%**	**%**	***P*****-value**^a^	**%**	**%**	**%**	***P*****-value**^a^
**GP visits for any reason 25–60 months pre-diagnosis (number of visits per year)**								
Median (IQR)		5 (2–10)	5 (2–10)	0.739		5 (2–9)	5 (2–12)	0.226
0 visits	12.1	12.9	10.5	0.756	12.1	12.7	9.2	0.124
1–2 visits	18.8	17.1	21.9		21.5	21.0	24.1	
3–4 visits	16.3	17.5	14.1		15.9	16.1	14.9	
5–9 visits	28.8	28.0	30.2		27.7	29.2	19.5	
10+ visits	24.1	24.6	23.3		22.7	21.0	32.2	
**GP visits for any reason 13–24 months pre-diagnosis (number of visits per year)**								
Median (IQR)		8 (3–14)	7 (3–13)	0.038		6 (2–11)	9 (4–15)	0.002
0 visits	6.0	9.1	7.1	0.056	8.4	6.9	8.2	0.002
1–2 visits	13.5	14.7	13.9		18.2	6.9	16.5	
3–4 visits	12.4	13.3	12.7		12.2	12.6	12.3	
5–9 visits	24.7	24.4	24.6		31.8	28.7	31.4	
10+ visits	43.4	38.5	41.7		29.4	44.8	31.7	
**GP visits for any reason between 30 days and 12 months pre-diagnosis**								
Median (IQR)		12 (7–18)	10 (5–19)	0.041		9 (5–13)	12 (6–20)	0.010
0 visits	2.4	2.1	3.1	0.008	3.1	3.1	3.5	0.068
1–2 visits	5.3	3.9	7.8		9.2	9.0	10.3	
3–4 visits	7.2	6.3	8.9		11.3	12.2	5.8	
5–9 visits	26.3	26.8	25.5		29.3	30.6	21.8	
10+ visits	58.8	60.9	54.9		47.1	45.1	58.6	

Abbreviations: EP=emergency presentation; GP=general practioner; IQR=interquartile range; non-EP=non-emergency presentation.

a The Wilcoxon rank-sum test was used for comparing median number of visits. Test for trend was calculated for categorical variable of GP visits.

**Table 3:** GP consulations with relevant symptoms for patients diagnosed with colon or rectal cancer following EP and non-EP by time before diagnosis

	**Colon cancer**				**Rectal cancer**			
	**Total**	**Non-EP**	**EP**		**Total**	**Non-EP**	**EP**	
	***N*****=1029**	***N*****=668**	***N*****=361**		***N*****=577**	***N*****=490**	***N*****=87**	
	**%**	**%**	**%**	***P*****-value**^a^	**%**	**%**	**%**	***P*****-value**^a^
**Patients with any relevant symptom**								
12 months pre-diagnosis	80.1	82.6	75.4	0.005	84.4	86.3	73.6	0.002
Between 30 days and 12 months pre-diagnosis	62.7	70.7	47.9	<0.001	59.3	61.0	49.4	0.043
30 days pre-diagnosis	37.9	29.8	52.9	<0.001	43.0	42.7	44.8	0.706
**No. of consultations with relevant symptoms between 30 days and 12 months pre-diagnosis**								
0 consultations	37.3	29.3	52.1	<0.001	40.7	39.0	50.6	0.094
1–2 consultations	42.9	48.8	31.9		43.2	44.9	33.3	
3+ consultations	19.8	21.9	16.1		16.1	16.1	16.1	
**At least one red-flag symptom (anaemia, rectal bleeding, change in bowel habits)**								
Between 30 days and 12 months pre-diagnosis	31.8	39.5	17.5	<0.001	36.4	38.8	23.0	0.005
**Specific symptoms recorded during the 30 days pre-diagnosis**								
Abdominal pain	15.7	8.7	28.8	<0.001	4.3	2.0	17.2	<0.001
Anaemia	6.2	7.9	3.1	0.002	3.0	3.1	2.3	0.698
Constipation	4.7	2.0	9.7	<0.001	4.0	2.9	10.3	0.001
Diarrhoea	4.2	2.3	7.8	<0.001	5.9	5.9	5.8	0.950
Rectal bleeding	4.4	5.1	3.1	0.126	17.2	19.0	6.9	0.006
Weight loss	1.8	1.7	1.9	0.733	1.7	1.2	4.6	0.026
Change in bowel habit	2.5	3.0	1.7	0.194	9.7	10.8	3.5	0.032
Fatigue	0.9	1.1	0.6	0.417	0.7	0.4	2.3	0.050
**Specific symptoms recorded between 30 days and 12 months pre-diagnosis**								
Abdominal pain	25.1	25.5	24.4	0.705	9.4	8.8	12.6	0.254
Anaemia	19.2	23.2	11.9	<0.001	6.2	5.9	8.1	0.450
Constipation	8.1	8.7	6.9	0.323	8.2	8.6	5.8	0.375
Diarrhoea	9.9	9.9	10.0	0.962	12.0	11.2	16.1	0.197
Rectal bleeding	9.4	12.6	3.6	<0.001	21.5	23.7	9.2	0.002
Weight loss	3.1	3.1	3.1	0.932	1.7	1.6	2.3	0.661
Change in bowel habit	5.5	6.7	3.3	0.022	11.6	12.2	8.1	0.260
Fatigue	4.4	4.9	3.3	0.226	2.3	2.5	1.2	0.452
**Specific symptoms recorded between 13–24 months pre-diagnosis**								
Abdominal pain	6.6	6.9	6.1	0.625	3.8	3.7	4.6	0.678
Anaemia	4.8	5.1	4.2	0.502	2.4	2.2	3.5	0.501
Constipation	3.7	3.9	3.3	0.645	1.4	1.2	2.3	0.430
Diarrhoea	2.7	3.1	1.9	0.257	3.3	3.7	1.2	0.224
Rectal bleeding	1.2	1.2	1.1	0.898	2.1	1.6	4.6	0.074
Weight loss	0.9	1.2	0.3	0.130	1.0	1.0	1.2	0.913
Change in bowel habit	0.2	0.2	0.3	0.658	0.2	0.2	0.0	0.673
Fatigue	2.4	2.3	2.8	0.602	1.4	1.4	1.2	0.837
**Specific symptoms recorded between 25–60 months pre-diagnosis**								
Abdominal pain	11.7	12.1	10.8	0.528	7.1	7.4	5.8	0.592
Anaemia	3.3	3.0	3.9	0.449	3.0	2.0	8.1	0.002
Constipation	5.5	5.5	5.5	0.999	3.3	2.9	5.8	0.164
Diarrhoea	6.1	5.4	7.5	0.182	4.7	4.5	5.8	0.609
Rectal bleeding	3.1	3.9	1.7	0.049	3.8	3.7	4.6	0.678
Weight loss	1.1	1.4	0.6	0.238	1.6	1.8	0.0	0.203
Change in bowel habit	1.6	1.8	1.1	0.394	0.7	0.4	2.3	0.050
Fatigue	3.3	3.9	2.2	0.151	3.6	3.7	3.5	0.918

Abbreviations: EP=emergency presentation; GP=general practioner; non-EP=non-emergency presentation.

a *χ*^2^-Test.

**Table 4:** Multivariable logistic regression OR for colon and rectal cancer diagnosed after EP compared with non-EP taking into account patient socio-demographic characteristics, number of GP consultations for any reason the year before diagnosis (excluding 30 days) and symptoms recorded in primary care (*N*=1606)

	**Colon Cancer**				**Rectal Cancer**			
	**OR**	**95% CI**		***P*****-value**	**OR**	**95% CI**		***P*****-value**
Gender								
Men	1				1			
Women	1.37	1.04	1.82	0.028	1.49	0.89	2.48	0.128
Age (years)								
25–59	1.09	0.68	1.74	0.721	0.47	0.16	1.34	0.158
60–69	1				1			
70–79	1.02	0.69	1.53	0.910	0.79	0.41	1.53	0.491
80+	1.84	1.24	2.73	0.002	1.40	0.75	2.62	0.286
Socio-economic deprivation quintile								
1 (least deprived)	1				1			
2	1.29	0.84	2.00	0.247	1.48	0.67	3.28	0.333
3	0.88	0.61	1.28	0.513	1.44	0.68	3.06	0.344
4	1.11	0.76	1.62	0.584	2.30	1.00	5.26	0.049
5 (most deprived)	1.50	0.92	2.45	0.106	3.47	1.50	8.03	0.004
No. of GP visits between 30 days and 12 months pre-diagnosis	1.00	0.99	1.01	0.658	1.03	1.01	1.06	0.008
Symptoms recorded between 30 days and 12 months pre-diagnosis								
Anaemia	0.38	0.26	0.55	<0.001	0.73	0.28	1.92	0.530
Change in bowel habits	0.47	0.25	0.87	0.017	0.60	0.26	1.41	0.241
Rectal bleeding	0.22	0.12	0.41	<0.001	0.25	0.11	0.58	0.001
Symptoms recorded between 25–60 months pre-diagnosis								
Anaemia	1.68	0.75	3.77	0.212	2.67	0.80	8.86	0.109
Change in bowel habits	0.73	0.21	2.50	0.617	11.96	1.55	92.09	0.017
Rectal bleeding	0.46	0.19	1.11	0.085	0.83	0.30	2.30	0.720

Abbreviations: CI=confidence interval; EP=emergency presentation; GP=general practioner; non-EP=non-emergency presentation; OR=odds ratio.
