# Supplementary material for: Do colorectal cancer patients diagnosed as an emergency differ from non-emergency patients in their consultation patterns and symptoms? A longitudinal data-linkage study in England
Source: Br J Cancer. 2016 Aug 18;115(7):866–75. doi: 10.1038/bjc.2016.250 (PMC5046207; doi:10.1038/bjc.2016.250)
Supplement: Supplementary Material 2 [file bjc2016250x2.pdf]

**Supp Mat 2: Development of a comprehensive Read codes list of relevant signs and symptoms for colorectal cancer**

- A preliminary list of signs/symptoms that could prompt diagnostic work-up for a possible colorectal cancer has been developed based on the review of the literature and clinical guidelines.
- The list of relevant signs/symptoms has been discussed and reviewed with clinical experts and project collaborators. In particular, experts and collaborators included three GPs with a specific interest in cancer; a consultant gastroenterologist with expertise in using CPRD; an oncologist with expertise in patient pathways; four public health specialists and epidemiologists with specific expertise in cancer diagnosis. Colorectal cancer patients have also taken part in discussing relevant signs/symptoms.
- Read codes for relevant signs/symptoms have been identified. We have applied the pre-specified list to the CPRD records; using the Read Code hierarchy and key-words we have expanded the code list including additional relevant Read codes.
